# Increased central auditory gain in 5xFAD Alzheimer’s disease mice as an early biomarker candidate for Alzheimer’s disease diagnosis

**DOI:** 10.3389/fnins.2023.1106570

**Published:** 2023-05-26

**Authors:** Daxiang Na, Jingyuan Zhang, Holly J. Beaulac, Dorota Piekna-Przybylska, Paige R. Nicklas, Amy E. Kiernan, Patricia M. White

**Affiliations:** ^1^Department of Biomedical Genetics, University of Rochester School of Medicine and Dentistry, Rochester, NY, United States; ^2^Department of Neuroscience, Ernest J. Del Monte Institute for Neuroscience, University of Rochester School of Medicine and Dentistry, Rochester, NY, United States; ^3^Department of Ophthalmology, University of Rochester, Rochester, NY, United States

**Keywords:** Alzheimer’s disease, central auditory gain, hearing loss, auditory brainstem response, central auditory processing disorder, hearing in noise, inhibitory deficit

## Abstract

Alzheimer’s Disease (AD) is a neurodegenerative illness without a cure. All current therapies require an accurate diagnosis and staging of AD to ensure appropriate care. Central auditory processing disorders (CAPDs) and hearing loss have been associated with AD, and may precede the onset of Alzheimer’s dementia. Therefore, CAPD is a possible biomarker candidate for AD diagnosis. However, little is known about how CAPD and AD pathological changes are correlated. In the present study, we investigated auditory changes in AD using transgenic amyloidosis mouse models. AD mouse models were bred to a mouse strain commonly used for auditory experiments, to compensate for the recessive accelerated hearing loss on the parent background. Auditory brainstem response (ABR) recordings revealed significant hearing loss, a reduced ABR wave I amplitude, and increased central gain in 5xFAD mice. In comparison, these effects were milder or reversed in APP/PS1 mice. Longitudinal analyses revealed that in 5xFAD mice, central gain increase preceded ABR wave I amplitude reduction and hearing loss, suggesting that it may originate from lesions in the central nervous system rather than the peripheral loss. Pharmacologically facilitating cholinergic signaling with donepezil reversed the central gain in 5xFAD mice. After the central gain increased, aging 5xFAD mice developed deficits for hearing sound pips in the presence of noise, consistent with CAPD-like symptoms of AD patients. Histological analysis revealed that amyloid plaques were deposited in the auditory cortex of both mouse strains. However, in 5xFAD but not APP/PS1 mice, plaque was observed in the upper auditory brainstem, specifically the inferior colliculus (IC) and the medial geniculate body (MGB). This plaque distribution parallels histological findings from human subjects with AD and correlates in age with central gain increase. Overall, we conclude that auditory alterations in amyloidosis mouse models correlate with amyloid deposits in the auditory brainstem and may be reversed initially through enhanced cholinergic signaling. The alteration of ABR recording related to the increase in central gain prior to AD-related hearing disorders suggests that it could potentially be used as an early biomarker of AD diagnosis.

## 1. Introduction

Alzheimer’s disease (AD) is a neurodegenerative illness most prevalent among the elderly. In 2021, it was estimated that over 55 million people worldwide live with dementia, of which AD is the most common (Gauthier et al., 2021). In the United States, the youngest baby-boomers will turn age 65 in 2028, and so AD diagnoses are expected to rapidly increase in the next decades (He et al., 2016). It is also estimated that COVID-19 contributed to a 17% increase in Alzheimer’s and dementia deaths in 2020 (Alzheimer’s Association, 2021). AD is a financial burden on the American public: in 2021, approximately $355 billion was spent to care for dementia patients. This does not include the unpaid care provided by families, which is estimated at $257 billion or more (Alzheimer’s Association, 2021). Today, there is no effective disease-modifying therapy available for AD. Early intervention could slow disease progression (Long and Holtzman, 2019), with significant impacts on cost-effectiveness (Barnett et al., 2014). Therefore, there is an urgent demand for new approaches to diagnose and track AD progression as early as possible.

Disordered auditory processing has been suggested as a promising biomarker candidate for AD diagnosis (Albers et al., 2015). A number of epidemiological studies suggest an association between hearing impairment and AD (Uhlmann et al., 1989; Lin et al., 2011, 2013; Amieva et al., 2015; Deal et al., 2017; Golub et al., 2017). Beyond peripheral hearing loss, studies in small cohorts also reported central auditory processing disorders (CAPDs) in AD patients [reviewed in Albers et al. (2015)]. AD patients showed difficulty in understanding speech in the presence of background noise (Ouchi et al., 2015), dichotic listening (Grimes et al., 1985; Mohr et al., 1990; Gates et al., 1995, 2008; Strouse et al., 1995; Idrizbegovic et al., 2011) and sound localization (Kurylo et al., 1993). Prospective studies reported that CAPD can precede the onset of AD dementia (Gates et al., 2002, 2011). Moreover, the diminished ability to detect gaps in noise clearly distinguished patients with mild cognitive impairment from normal controls and correlated with temporal cortical thinning (Iliadou et al., 2017a).

There is evidence suggesting that CAPD in AD may be associated with inhibitory deficits. Central auditory hyperactivity, which has been attributed to inhibitory loss (Parthasarathy et al., 2019), was reported in patients diagnosed with mild cognitive impairment (Irimajiri et al., 2005). Pharmacologically facilitating the transmission of acetylcholine, which plays critical roles in modulating neural activity at multiple levels of the auditory system (Schofield et al., 2011), may attenuate this hyperactivity. Similar treatment can also reduce the deficit for hearing in noise seen in AD patients (Ouchi et al., 2015).

The underlying relationship between CAPDs and AD requires further investigation. Could central auditory hyperactivity be a biomarker for AD diagnosis? Unlike many central auditory processing functions, central hyperactivity can be assessed by non-behavioral tests in rodents, which can minimize the interference caused by AD-related motor deficits in 5xFAD models (Jawhar et al., 2012). Auditory event related potentials (auditory ERPs) are obtained from the auditory brainstem response (ABR) to tones or clicks. Central gain is calculated by comparing the amplitude of a later potential, or wave, to that of the first potential, which is derived from the cochlear nerve. Greater relative amplitudes in later waves are a demonstration of central hyperactivity. In the present study, we compared two major models for investigating hearing loss and CAPDs in AD: the 5xFAD and APP/PS1 transgenic amyloidosis mouse models. Both models were bred on the same CBA/B6 hybrid background to control for strain effects. By recording auditory functions and analyzing plaque distribution in these mice, we investigated how hearing loss and CAPD correlate with AD progression. Through histological analysis, we further correlated the locations of amyloid plaque deposition in the auditory pathway with the onset of central gain.

## 2. Methods

### 2.1. Mice

5xFAD (Oakley et al., 2006) and APP/PS1 (Jankowsky et al., 2004) mouse lines with C57BL/6 J congenic background were obtained from the Jackson Laboratory (Bar Harbor, ME, USA; stock no. 34848-JAX and 34,832-JAX). We sought to compensate the known hearing loss deficit in C57BL/6 J (Noben-Trauth et al., 2003) by breeding those lines to wild-type CBA/CaJ, to obtain heterozygote mutant mice and their wild-type (WT) littermates with a CBA/B6 hybrid background. To eliminate effects of amyloidosis on maternal care, transgenic males (5xFAD or APP/PS1) were bred to CBA/CaJ females. To ensure the consistency in genetic background, only F1 heterozygote 5xFAD males, heterozygote APP males, and their WT male littermates were used in this study. For mouse genotyping, DNA obtained from 2-mm tail samples were processed and verified by Transnetyx Automated PCR Genotyping Services (Transnetyx, Inc. Cordova, TN, USA).

Mice were housed on a 12-h light/dark cycle and received chow and water *ad libitum*. The appearance of new litters was compared with the Jackson Laboratory pups’ age appearance chart and their birth date was designated postnatal day (P) 0. Mice were weaned between P21-28 and no more than five same sexed adults were housed per cage. They were provided ample nesting materials and small houses within their home cages. Using the NIOSH Sound Level Meter app (Centers for Disease Control, Washington, DC, USA), ambient noise in the cage interior centered around 58 dB in the 20 Hz to 20 kHz range. Cages to be used for experiments were maintained in the center of the holding rack to avoid excess noise.

### 2.2. Auditory brainstem response and distortion product otoacoustic emissions

Hearing thresholds were tested following the schedules as described in the text. Testing occurred between the hours of 9 am–6 pm. Mice were anesthetized with a single intraperitoneal (i.p.) injection of ketamine (Hikma, 0143-9509-01, 80 mg/kg animal weight) and acepromazine (Vet One, 13985-587-50, 3 mg/kg animal weight) diluted in a sterile saline solution, to provide approximately 45 min of immobility. Additional anesthetic was administered as needed. A 10B+ OAE microphone was housed in an interaural probe and coupled with the speaker outputs. The probe was placed at the opening of each mouse’s left external auditory meatus. A Smart EP Universal Smart Box (Intelligent Hearing Systems, Miami, FL, USA) with an ED1 speaker (Tucker Davis Technologies, Alachua, FL, USA) were housed within an anechoic chamber and used for closed field auditory testing. The Quest Technologies portable sound level meter, Model 1900 (TSI Incorporated, Shoreview, MN, USA) was used to calibrate the apparatus less than one month prior to beginning each set of experiments.

The ABR was recorded as described in previous studies (Gilels et al., 2013; Beaulac et al., 2021). Both, click-evoked and pure tone-evoked ABRs were recorded. The ABRs consisted of 50-microsecond (μs) click stimuli or 1-millisecond (ms) tone pips presented at five frequencies (8, 12, 16, 24, and 32 kHz). Stimuli amplitudes decreased in 5 decibel (dB) steps from 75 dB sound pressure level to 15–25 dB (5 dB for click stimuli). The averages of 512 sweeps were recorded for each frequency and amplitude. Three sterilized fine subdermal electrodes were used to record electrical responses (Grass): one inserted at the vertex and one inserted beneath each pinna. Responses were rejected if their peak to trough amplitude was greater than 31 microvolts (μV) at any time between 1.3 to 12.5 ms after stimulus presentation. Well-anesthetized mice typically had a 5–30% rejection rate. ABR thresholds were determined by the last visible trace of wave I (dB). If no waveform was observed, “80 dB” was designated as the ceiling threshold.

ABR peaks and troughs were registered by a blinded reviewer with custom scripts^1^ (Na and White, 2022). Wave latency was defined as the difference between auditory stimuli onset (0 ms) to the time of the peak apex (ms). Wave amplitude was defined as the difference between the peak apex and the following trough (μV). In this study, wave I (peak 1 or p1) and wave IV (p4) latency and amplitude were extracted, wave IV to I amplitude ratio (p4:p1) and wave I to IV interpeak latency (p1–p4 interval) were calculated.

Distortion product otoacoustic emissions (DPOAE) were measured using the amplitude of evoked otoacoustic emissions to simultaneous pure tones of frequencies f_1_ and f_2_, where f_1_/f_2_ = 1.2 and the f_1_ level is 10 dB above f_2_. Beginning with f_1_ at 20 dB and ending at 65 dB, 32 sweeps were made in 5 dB steps. The DPOAE threshold was calculated for 3 dB emission. As a second calibration, measurements were collected from a dead mouse and L_2_ amplitudes with signals above threshold were excluded.

For the experiment in which the ABR stimuli were masked with background noise, a Duet system (Intelligent Hearing Systems) with two ED1 speakers (Tucker Davis Technologies) was used. With the additional speaker, a broadband noise (masking) or a broadband noise with an additional 1 octave spectral notch (notched) was included throughout the entire presentation of the ABR stimuli. ABR stimuli were 5-ms tone pips at 8 kHz. Stimuli amplitudes decreased in 5 dB steps from 100 dB sound pressure level to 25 dB. ABR with masking noise is denoted as “masking” and notched noise as “notched”. The ABR without noise was recorded within the same session. Threshold shift was calculated by subtracting ABR threshold with noise from ABR threshold without noise. For 5xFAD mice used in this experiment, only the mice with normal hearing thresholds were used to minimize the effect caused by hearing loss. Normal hearing thresholds were defined as the thresholds within 2 standard deviations of the WT littermates’ ABR threshold without noise.

After completing auditory testing, anesthetized mice were isolated in recovery cages until they woke up. Their arousal levels were monitored, and mice were returned to their home cages after they regained consciousness. The researchers scoring ABRs and DPOAEs were blinded to genotype, condition, and time point.

### 2.3. Noise exposure

Mice at 3.5 months of age (P110, denoted as 3.5 M) were exposed to an 8–16 kHz octave noise band at 105 dB for 30 min. Awake mice were placed in individual triangular wire mesh cages, 12 cm × 5 cm × 5 cm, in an asymmetric plywood box with a JBL2250HJ compression speaker and JBL2382A biradial horn mounted above. This apparatus was contained within a sound booth. The speaker was driven by a TDT RX6 multifunction processor and dedicated attenuator (Tucker Davis Technologies). It was controlled with TDT RPvdsEx sound processing software (Tucker Davis Technologies). All noise exposure equipment was calibrated prior to each use *via* the Quest Technologies portable sound level meter, Model 1900 (TSI Incorporated). Within the sound exposure box, cages were placed in three specific locations where sound levels were highly consistent (± < 0.5 dB). Mice that escaped or moved their cages from the starting position were excluded. Mice were directly monitored for the first minute of exposure through the plexiglass window on the chamber. Due to known circadian cycle interactions with noise damage, mice were exposed to noise only between the hours of 9 am and 1 pm (Meltser et al., 2014). After noise exposures, mice were monitored for proper health status and closely examined up until sacrifice. Mice exhibiting signs of pain or distress were euthanized early and excluded from further analysis. The ABR and DPOAE threshold shift is defined as the difference between the threshold at P90 and post noise exposure.

### 2.4. Donepezil treatment

5xFAD (*n* = 6) and WT littermate (*n* = 4) male mice at 6 months of age were used. Mice were treated with donepezil at 1 mg*kg^−1^*day^−1^. Donepezil hydrochloride (MedChemExpress, Monmouth Junction, NJ, USA) was dissolved in water with 1.8% 2-hydroxypropyl-β-cyclodextrin and administered through drinking water. During treatment, donepezil was freshly dissolved in water each week. Prior to treatment and after 4 weeks of treatment, ABR and DPOAE tests were performed to evaluate auditory function, following the same procedure described in Section 2.2.

### 2.5. Histological quantification of amyloid plaques

Histological analysis was performed on brains of 5xFAD mice aged 3 months (3 M), 6 M, and 12 M; APP/PS1 mice aged 13 M, and WT littermates aged 12 M (*n* = 6 for each age and genotype). Briefly, brains were dissected from mice immediately after perfusion with saline and 4% paraformaldehyde and further immersed in 4% paraformaldehyde for 2 hours at 4°C. After this, brains were washed once with PBS and then stored in PBS at 4°C until processing. The brains were embedded in agarose (Precisionary Instruments LLC, Natick, MA, USA) and 80-micron coronal sections were cut on a compresstome (Precisionary Instruments LLC). Sections were collected and stored in 24-well plates in PBS at 4°C, with care to preserve their order for later reference to their stereotaxic coordinates.

Sections containing the brain nuclei of interest were selected by comparison with the Mouse Brain Atlas (Franklin and Paxinos, 2019). Specifically, sections containing the subiculum (Sub) and CA1 region of the hippocampus were selected as positive controls for amyloid deposition. Regions of the auditory cortex (AC), the medial geniculate nucleus (MGB), the inferior colliculus (IC), the medial nucleus of the trapezoid body (MNTB), the superior olivary complex (SOC), and the cochlear nucleus (CN) were analyzed for potential correlates to auditory processing disorder. Floating sections were stained overnight at 4°C with 6E10 (1:3000, Biolegend cat#803014, RRID: AB_2564657) diluted in PBS with 5% donkey serum to label amyloid plaque. They were washed three times for 30 min in PBS with gentle rocking, further incubated with donkey anti-mouse antibodies conjugated to Alexa 594 (1:500, Jackson Immunoresearch, 715-606-150) and Neurotrace 500/525 (1,500, ThermoFisher #N21480) at room temperature for two hours, and washed three times for 30 min in PBS. Tissues were mounted in Fluoromount-G (Southern Biotech cat#0100-01) and cover slipped on microscope slides.

For each animal, 3–4 coronal tissue sections that included the Sub, CA1, AC, MGB, IC, CN, SOC, and MNTB were imaged with a Leica Stellaris 5 Inverted Confocal Microscope using a 5× or 10× objective lens as indicated in the figure legends. Imaging parameters were kept constant across all sections for each set of immunofluorescent labels. Experimenters were blinded to the genotypes and ages of the animals. All the images were processed with ImageJ FIJI (NIH) and analyzed with a custom CellProfiler (Stirling et al., 2021) pipeline.

For the plaque coverage analysis, regions of interest (ROIs) outlining the above-mentioned structures were drawn on maximum z-projections of the acquired 6E10 images and the corresponding masks were generated with a custom ImageJ plugin. Images were subsequently thresholded and binarized using the minimum cross-entropy thresholding algorithm embedded in CellProfiler. The plaque coverage was calculated as the ratio between the number of pixels thresholded over all pixels in the ROIs.

### 2.6. Whole-brain plaque deposition datasets

Some data analyses presented are based on a publicly available dataset (Oblak et al., 2021), which registered plaque deposition using the Allen Mouse Brain Common Coordinate Framework (Wang et al., 2020). Mice were given an intraperitoneal injection of methoxy-X04 to label amyloid plaques. Whole-brain serial two-photon (STP) tomography imaging was performed using a TissueCyte 1000 (TissueVision, Inc.) Plaque density, which is defined as the plaque volume divided by structure volume, was calculated in the study. In the present analysis, data from 5xFAD males on congenic C57BL/6 J background at ages 2 M, 3 M, 4 M, and 6 M were analyzed.

### 2.7. Statistical analysis

Sample sizes were calculated for sufficient power analysis. The sample size for each experiment is indicated within each figure legend. The researcher was blinded as to the genotype and condition for the ABR and DPOAE threshold scoring and ABR waveform analyses. The Shapiro Wilk test was used to assess normality for each data set, and nonparametric tests were used for all non-normal datasets. Unpaired student’s *t*-tests, Mann–Whitney U rank sum test, two-way ANOVA, and Kruskal-Wallis tests were used to compare differences across groups. Bonferroni adjustment and the Holm-Šídák multiple comparisons test were used for post-hoc analysis. Tests used for each experiment are indicated in the text. A value of *p* <0.05 was considered statistically significant. All data are presented as group mean with standard error of the mean (SEM). For ABR waveform analyses (amplitude, latency, amplitude ratio), group mean is presented as the thick line with SEM as shading and individual data as thin traces. For plaque accumulation, individual data are presented as dots. In all figures, the *p*-values are defined as: no significance (n.s.), *p* ≥ 0.05; * *p* < 0.05; ** *p* < 0.01, and *** *p* < 0.001. Statistics and data plotting were performed in GraphPad Prism 9.3.1 and R 4.1.2.

## 3. Results

### 3.1. 5xFAD transgenic mice have increased central gain and hearing loss severity

Auditory changes including hearing loss and gap detection deficits have been characterized in several transgenic AD mouse models including 5xFAD and APP/PS1 (O’Leary et al., 2017; Kaylegian et al., 2019; Liu et al., 2020; Weible et al., 2020; Mei et al., 2021). To test if increased central gain occurs in AD mouse models, we performed auditory tests in 5xFAD and APP/PS1 mice at similar ages (5xFAD at 12 months of age and APP/PS1 at 13 months of age), by which time both of the strains have significant plaque accumulation (Jankowsky et al., 2004; Oakley et al., 2006). To compensate for accelerated hearing loss on the C57BL/6 J background, transgenic C57BL/6 J males were bred to wild-type CBA/CaJ females. To ensure consistency in their genetic background, only F1 mice were used in the present study.

**Figure 1:**
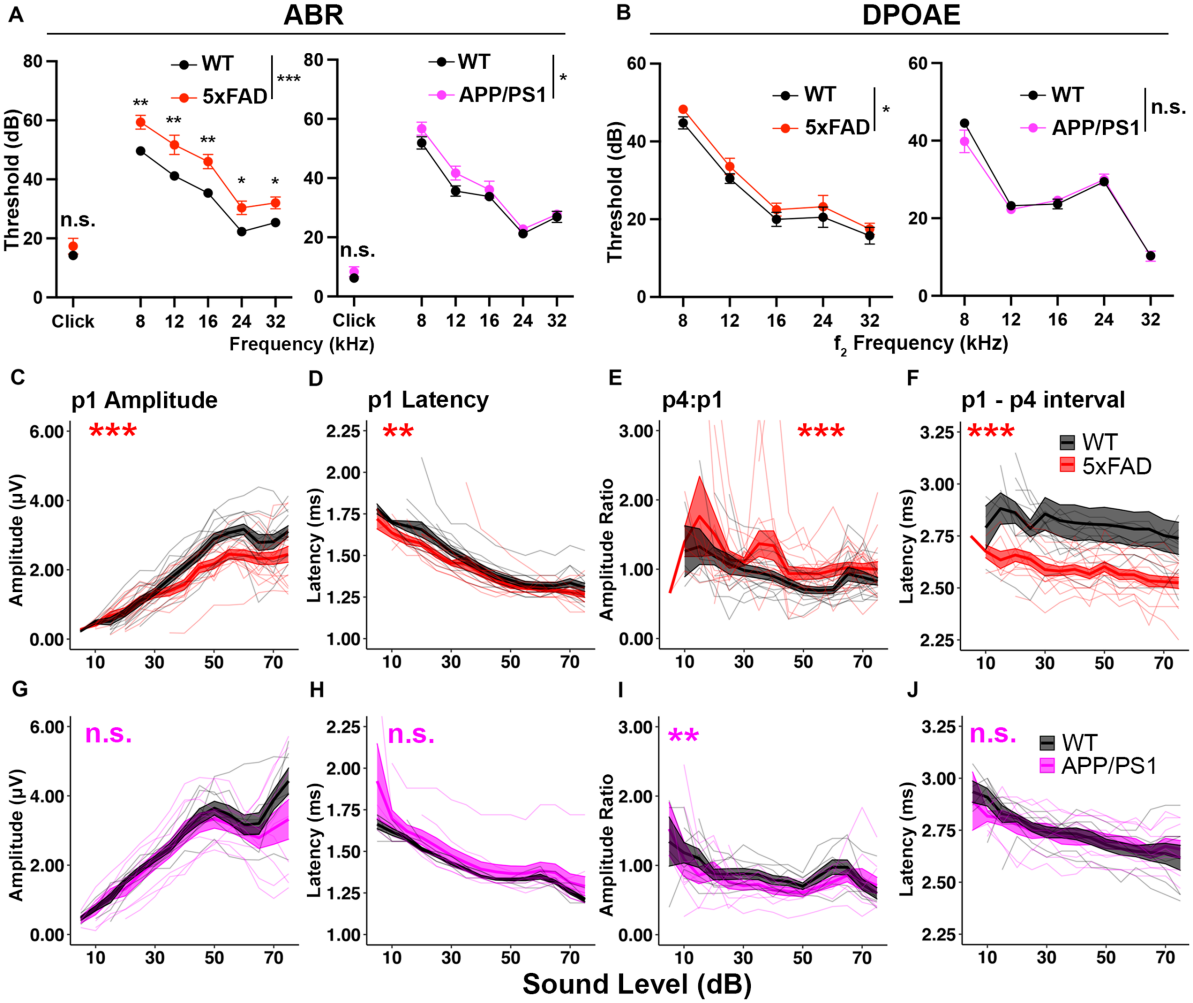
5xFAD transgenic mice have increased central gain and hearing loss severity. Auditory test results of **(A–F)** 5xFAD (red) at 12 months of age (12M) (WT *n* = 13, 5xFAD *n* = 15), and **(G–J)** for APP/PS1 (magenta) at 13M (WT *n* = 8, APP/PS1 *n* = 9). **(A–J)** Corresponding wild-type (WT) littermate data (black). **(A)** ABR and **(B)** DPOAE thresholds are expressed as the mean ± SEM. **(C–J)** Wave I (p1) amplitude, latency, wave IV to I amplitude ratio (p4:p1) and wave I to wave IV interpeak latency of click-evoked ABRs. Asterisks denote significant differences between genotypes: no significance (n.s.), *p* ≥ 0.05; *p < 0.05; **p < 0.01; and ***p < 0.001.

AD pathology and auditory functions in mice can differ among genetic backgrounds. The AD mouse models with a CBA/B6 hybrid background have not been characterized in previous studies. To test if characterized hearing loss (O’Leary et al., 2017; Liu et al., 2020) can be reproduced in AD mice with a CBA/B6 hybrid background, we analyzed the ABR and DPOAE thresholds of those mice. ABR thresholds were significantly increased in aged 5xFAD mice, comparing to their WT littermates (Figure 1A, left, *p* < 0.0001 for pure tones, two-way ANOVA; *p* = 0.4147 for click, Mann–Whitney U rank sum test). Post-hoc analysis revealed significant differences at all frequencies tested in pure-tone evoked ABR thresholds (adjusted-*p* = 0.0031 for 8 kHz, adjusted-*p* = 0.0018 for 12 kHz, adjusted-p = 0.0018 for 16 kHz, adjusted-*p* = 0.0129 for 24 kHz and adjusted-*p* = 0.0241 for 32 kHz, Holm-Šídák multiple comparisons test). APP/PS1 mice also showed significant hearing loss in pure tone-evoked ABR thresholds (Figure 1A, right, *p* = 0.012 for pure tones, two-way ANOVA; *p* = 0.2615 for click, Mann–Whitney U rank sum test). However, changes were not significant at individual frequencies in the post-hoc analysis (adjusted-*p* = 0.2886 for 8 kHz, adjusted-*p* = 0.137 for 12 kHz, adjusted-*p* = 0.7699 for 16 kHz, adjusted-*p* = 0.8196 for 24 kHz and adjusted-*p* = 0.8196 for 32 kHz, Holm-Šídák multiple comparisons test). DPOAE thresholds were significantly increased in 5xFAD mice (Figure 1B, *p* = 0.0293, two-way ANOVA; adjusted-*p* = 0.0972 for 8 kHz, adjusted-*p* = 0.9817 for 12 kHz, adjusted-*p* = 0.9817 for 16 kHz, adjusted-*p* = 0.9817 for 24 kHz and adjusted-*p* = 0.9817 for 32 kHz, Holm-Šídák multiple comparisons test), but remained unchanged in APP/PS1 mice (Figure 1B, *p* = 0.3368, two-way ANOVA). Taken together, we conclude that both of 5xFAD and APP/PS1 mice on a CBA/B6 background show significant hearing loss. Hearing loss appeared more pronounced in 5xFAD mice.

To determine if auditory ERPs are relatively increased in AD mice, we analyzed the ABR waves I and IV to click stimuli. In 5xFAD mice, the wave I amplitude, which is the neuronal output from auditory nerves, was significantly reduced (Figure 1C, *p* = 8.679×10^−4^, Kruskal-Wallis test). The wave I latency was slightly but significantly reduced (Figure 1D, *p* = 2.164×10^−3^, Kruskal-Wallis test). The wave IV to wave I amplitude ratio (p4:p1), which has been used to evaluate central gain (Lowe and Walton, 2015; Möhrle et al., 2016; Schrode et al., 2018), was significantly increased (Figure 1E, *p* = 1.307×10^−6^, Kruskal-Wallis test). The wave I – IV interval latency was significantly shortened (Figure 1F, *p* < 2.2×10^−16^, Kruskal-Wallis test). The relative increase in auditory ERPs indicates increased central gain for 5xFAD.

In APP/PS1 mice, the wave I amplitude did not differ from their WT littermates (Figure 1G, *p* = 0.2007, Kruskal-Wallis test). The wave I latency also remained unaffected (Figure 1H, *p* = 0.193, Kruskal-Wallis test). The wave IV to wave I amplitude ratio was slightly reduced (Figure 1I, *p* = 4.684×10^−3^, Kruskal-Wallis test). Wave I to IV interpeak latency (p1–p4 interval) remained unaffected in the APP/PS1 mice (Figure 1J, *p* = 0.6961, Kruskal-Wallis test).

Overall, we conclude that the hearing loss phenotype was reproduced in both of 5xFAD and APP/PS1 mice, with a more severe phenotype in 5xFAD mice. Significant relative increases in auditory ERPs were observed in 5xFAD, but not APP/PS1, mice. These results supported further investigation of AD related increased central gain and its temporal relationship to other auditory deficits in the 5xFAD mouse model.

### 3.2. Auditory brainstem function is normal in young adult 5xFAD mice

**Figure 2:**
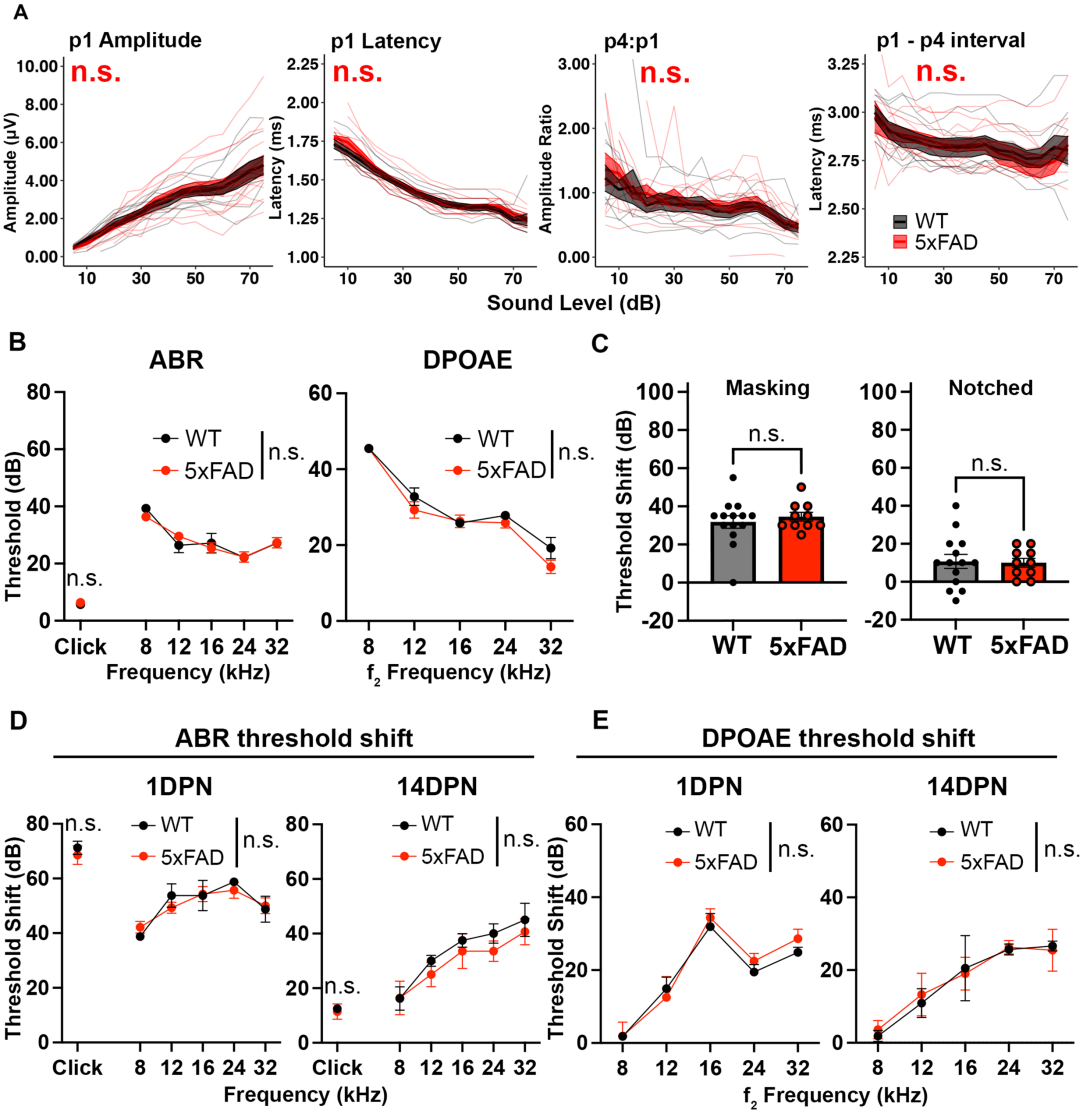
Auditory functions are normal in 5xFAD mice at an early stage of amyloid deposition. 5xFAD mice (red) and their WT littermates (black). **(A)** Wave I amplitude, latency, wave IV to I amplitude ratio, wave I to IV interpeak latency (from left to right) of click-evoked ABRs from 5xFAD mice (*n* = 14) and their WT littermates (*n* = 9) at 3M. **(B–E)** Data are expressed as the mean ± SEM. **(B)** ABR (left) and DPOAE (right) thresholds from 5xFAD mice (*n* = 11) and their WT littermates (*n* = 7) at 3M. **(C)** ABR threshold shift with masking and notched noise for 5xFAD mice (*n* = 10) and their WT littermates (*n* = 14) at 3M. **(D)** ABR and **(E)** DPOAE threshold shifts at 1 day (1 DPN) and 14 days (14 DPN) post noise exposure for 5xFAD mice (*n* = 7) and their WT littermates (*n* = 4) at 3.5M. No significance (n.s.).

In 5xFAD mice, significant amyloid deposition is seen in cortex and hippocampus at 2–3 months (M) of age (Oakley et al., 2006). Since aged 5xFAD mice show increased central activity and hearing loss, we asked if these phenotypes arise in conjunction with the timing of significant amyloid accumulation. To answer this question, we assessed ABRs in young adult 5xFAD mice at 3 M. The change of wave I amplitude in 5xFAD was insignificant at this age (Figure 2A, p1 amplitude, *p* = 0.9913, Kruskal-Wallis test). No change was observed in the wave I latency (Figure 2A, p1 latency, *p* = 0.6246, Kruskal-Wallis test). Differences between 5xFAD mice and WT littermates in wave IV to I amplitude ratio and interpeak latency change also remained insignificant (Figure 2A, *p* = 0.06542 for p4:p1 and *p* = 0.1159 for p1 to p4 interval, Kruskal-Wallis test). No significant differences between 5xFAD mice and WT mice in the ABR and DPOAE thresholds were identified (Figure 2B, *p* = 0.8425 for pure tone-evoked ABR threshold, two-way ANOVA; *p* = 0.8427 for click-evoked ABR threshold, two-way ANOVA; *p* = 0.0728 for DPOAE threshold, two-way ANOVA). At 3 M, we detected no change in central auditory activity and hearing threshold of 5xFAD mice.

Deficits for hearing in noise can arise in AD patients with normal audiograms (Albers et al., 2015). To evaluate hearing in noise in 5xFAD mice, we recorded ABRs evoked by tone pips in background noise with a spectral notch at 3 M. Noise interference lessens as the width of the notch increases (Patterson, 1974). Therefore, we performed the test with two levels of noise interference: masking and notched noise. With the addition of background noise, hearing thresholds shifted in these mice. The threshold shift was not significantly different between 5xFAD mice and their WT littermates (Figure 2C, *p* = 0.8037 for masking noise, Mann–Whitney U rank sum test; *p* = 0.8838 for notched noise, unpaired *t*-test). These results indicate that hearing in noise remains normal in 3 M 5xFAD mice.

In addition to receiving neural output from the cochlea and processing information, the brainstem protects the cochlea from age-related degeneration and noise damage *via* efferent synapses on OHCs (Maison et al., 2013; Liberman et al., 2014). To further evaluate brainstem function in the 5xFAD mice, we challenged mice at 3.5 M with a moderate noise exposure. The ABR threshold shift was evaluated at 1 day post noise exposure (1 DPN) and 14 DPN. Threshold shifts were not significantly different in 5xFAD mice at either time point (Figure 2D, 1 DPN, *p* = 0.8179 for pure tone-evoked ABR, two-way ANOVA; *p* = 0.8636 for click-evoked ABR, Mann–Whitney U rank sum test; 14 DPN, *p* = 0.3497 for pure tone-evoked ABR, two-way ANOVA; *p* = 0.6485 for click-evoked ABR, Mann–Whitney U rank sum test). DPOAE threshold shifts evaluated in conjunction with the ABRs did not show significant change between the 5xFAD mice and their WT littermates (Figure 2E, 1 DPN, *p* = 0.5534; 14 DPN, *p* = 0.8992, two-way ANOVA). Overall, we conclude that auditory function in young adult 5xFAD mice is indistinguishable from control littermates.

### 3.3. Relative increases in auditory ERPs precede both ABR wave I amplitude reduction and hearing loss in 5xFAD mice

**Figure 3:**
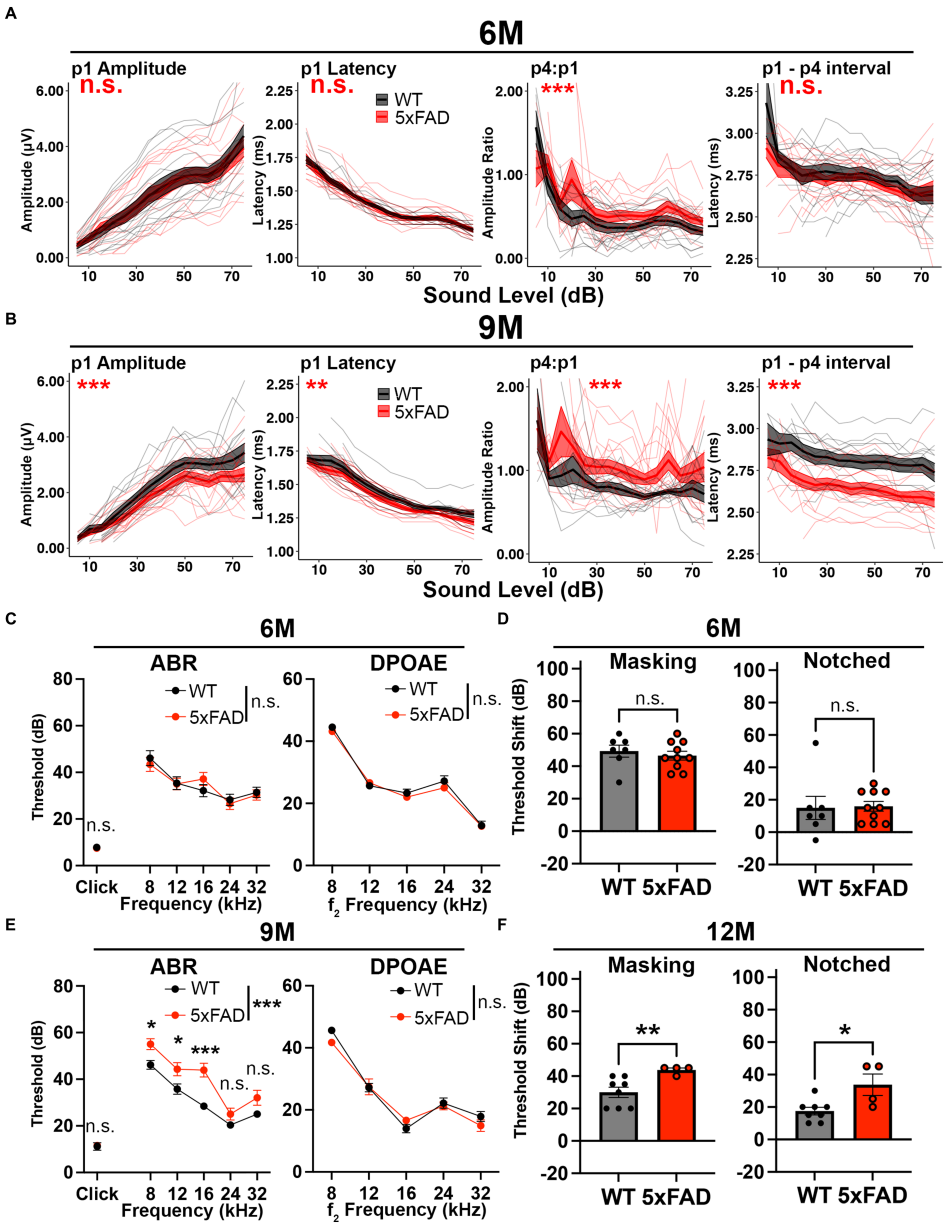
Central gain increases prior to central auditory processing disorder in 5xFAD mice. 5xFAD mice (red) and their WT littermates (black). **(A,B)** The wave I amplitude, latency, wave IV to I amplitude ratio, wave I to IV interpeak latency (from left to right) of click-evoked ABRs from 5xFAD (*n* = 16) and their WT littermates (*n* = 14) at **(A)** 6 M and **(B)** 9 M. **(C,E)** ABR (left) and DPOAE (right) thresholds of 5xFAD mice and their WT littermates at **(C)** 6 M and **(E)** 9 M. **(D,F)** ABR threshold shifts with masking (left) and notched noise (right) for 5xFAD mice and their WT littermates at **(D)** 6 M (5xFAD, *n* = 10; WT, *n* = 7) and **(F)** 12 M (5xFAD, *n* = 4; WT, *n* = 8). **(C–F)** Data are expressed as the mean ± SEM. Asterisks denote the significant differences between genotypes: no significance (n.s.), *p* ≥ 0.05; *p < 0.05; **p < 0.01; and ***p < 0.001.

Central hyperactivity has been observed as the reaction of the central auditory system to decreased auditory nerve output in situations such as noise damage, driving relative increases in later auditory ERPs (Parthasarathy et al., 2019). We analyzed how this increase in central gain progressed during aging in the 5xFAD mice. We performed auditory tests in 5xFAD mice at 6 M and 9 M. At 6 M, the wave I amplitude remained unchanged in 5xFAD mice (Figure 3A, p1 amplitude, *p* = 0.9429, Kruskal-Wallis test). The central gain was significantly increased in 5xFAD mice compared to their WT littermates (Figure 3A, p4:p1, *p* = 3.981×10^−6^, Kruskal-Wallis test). Changes of the wave I latency (p1 latency) and wave I to IV interpeak latency (p1 – p4 interval) were insignificant in 5xFAD mice (Figure 3A, *p* = 0.7882 for p1 latency, *p* = 0.07499 for p1–p4 interval, Kruskal-Wallis test).

Increased relative auditory ERPs persisted in 5xFAD at 9 M (Figure 3B, p4:p1, *p* = 2.177×10^−9^, Kruskal-Wallis test). At this time point, the wave I amplitude reduction became significant in 5xFAD mice (Figure 3B, p1 amplitude, *p* = 8.019×10^−4^, Kruskal-Wallis test). Starting at this age, the wave I latency became significantly shorter in 5xFAD mice than in WT mice (Figure 3B, p1 latency, *p* = 1.793×10^−3^, Kruskal-Wallis test), along with the wave I to IV interpeak latency (Figure 3B, p1 – p4 interval, *p* < 2.2×10^−16^, Kruskal-Wallis test).

At 6 M, 5xFAD mice did not show significant ABR and DPOAE threshold changes (Figure 3C, *p* = 0.9322 for pure tone-evoked ABR, two-way ANOVA, *p* = 0.6139 for click-evoked ABR, Mann–Whitney U rank sum test; *p* = 0.2976 for DPOAE, two-way ANOVA). With the presence of masking noise and notched noise, the ABR threshold shifts were unchanged when comparing 5xFAD mice to WT littermates (Figure 3D, *p* = 0.5409 for masking noise, unpaired *t* test; *p* = 0.5604 for notched noise, Mann–Whitney U rank sum test).

At 9 M, hearing loss became significant in 5xFAD mice (Figure 3E, *p* < 0.0001 for pure tone-evoked ABR, two-way ANOVA; *p* = 0.8517 for click-evoked ABR, Mann–Whitney U rank sum test). Post-hoc analysis showed that changes were significant at low frequencies (Figure 3E, adjusted-*p* = 0.0163 for 8 kHz, adjusted-*p* = 0.0193 for 12 kHz, adjusted-*p* < 0.0001 for 16 kHz, adjusted-*p* = 0.1714 for 12 kHz and adjusted-*p* = 0.0766 for 32 kHz, Holm-Šídák multiple comparisons test). These results are similar to a previous study, which showed a greater mean difference at low frequencies (8 kHz and 16 kHz) than at the high frequency (32 kHz) between 5xFAD and WT mice (O’Leary et al., 2017).

We further evaluated the hearing in noise in aged 5xFAD mice. For technical reasons, we were unable to perform ABR in masking and notched noise tests for the mice at 9 M. However, when we compared mice with equivalent 8 kHz ABR thresholds at 12 M, we found that threshold shifts for 5xFAD mice were significantly higher than their WT littermates in masking and notched noise (Figure 3F, *p* = 0.0081 for masking noise, *p* = 0.0364 for notched noise, Mann–Whitney U rank sum test). Together with the data in Figure 3D, these results indicate that hearing in noise became defective in 5xFAD mice after the onset of the central gain increase.

Taken together, we conclude that relative increases in later auditory ERPs preceded ABR wave I amplitude reduction and hearing loss in 5xFAD mice, which suggests that it may originate from lesions in the central nervous system rather than from peripheral loss. We also report a deficit for hearing in noise for the first time in 5xFAD mice.

### 3.4. Relative increases in auditory ERPs are caused by a cholinergic deficit in 5xFAD mice

**Figure 4:**
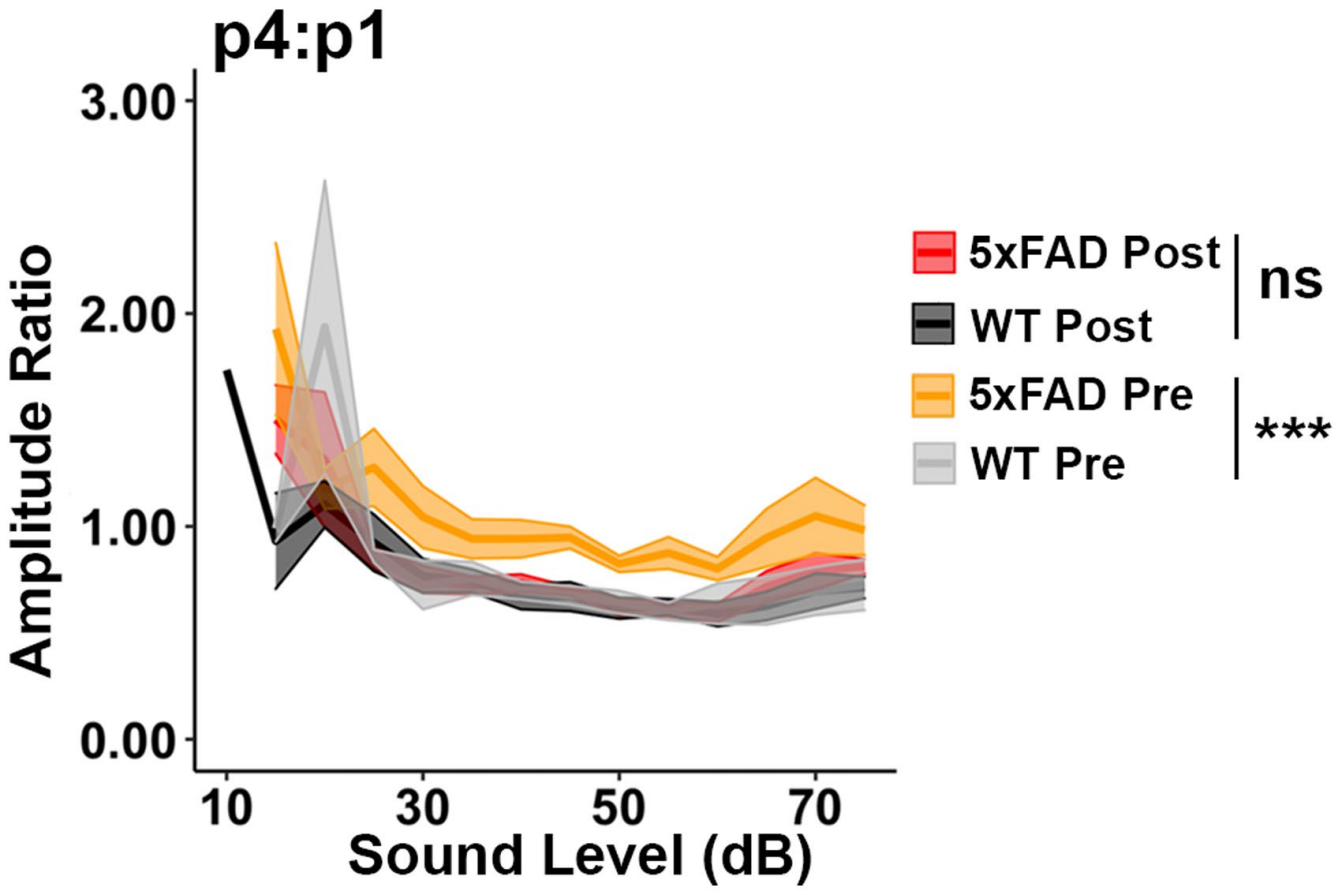
Increased central gain in 5xFAD mice is diminished after donepezil treatment. The wave IV to I amplitude ratio of click-evoked ABRs from 5xFAD mice (*n* = 6) and their WT littermates (*n* = 4) before (Pre, yellow 5xFAD, gray WT) and after (Post, red 5xFAD, black WT) donepezil treatment. Asterisks denote the significant differences between genotypes: no significance (n.s.), *p* ≥ 0.05; *p < 0.05; **p < 0.01; and ***p < 0.001.

Donepezil treatment is commonly applied in AD treatment, as it can improve cognition when patients are experiencing mild to moderate AD (Long and Holtzman, 2019). Donepezil enhances the transmission of acetylcholine by inhibiting activity of acetylcholinesterase (Bryson and Benfield, 1997). To test if the relative increases in auditory ERPs in 5xFAD mice originate from a cholinergic defect, we evaluated the change in ABR wave IV to I amplitude ratio before and after donepezil treatment. Prior to donepezil treatment, the wave IV to I amplitude ratio (p4:p1) was significantly higher in 5xFAD mice at 6 M (Figure 4, WT Pre vs. 5xFAD Pre, *p* < 0.0001, Kruskal-Wallis test), consistent with our observation in Figure 2. After 4 weeks of treatment, the p4:p1 difference was insignificant (Figure 4, WT Post vs. 5xFAD Post, *p* = 0.2782, Kruskal-Wallis test). The difference in wave I amplitude between WT and 5xFAD mice was not significant before and after treatment (Supplementary Figure S1A, WT Pre vs. 5xFAD Pre, *p* = 0.3379; Supplementary Figure S1B, WT Post vs. 5xFAD Post, *p* = 0.2696, Kruskal-Wallis test). Overall, we conclude that soon after it is established, the increased central gain in 5xFAD mice can be attenuated by pharmacologically facilitating cholinergic signaling.

### 3.5. Amyloid plaque accumulation in the central auditory pathway is significant in 5xFAD mice

Given that abnormal central auditory activity was observed in 5xFAD mice at 6 M, we hypothesize that AD-related pathology occurs in the central auditory pathway at this stage. The auditory cortex (AC) is one region where amyloid plaques are frequently identified in AD patients (Sinha et al., 1993). Considering that the ABR reflects activity in the auditory brainstem, we also analyzed plaque accumulation at multiple levels of the auditory brainstem, including the medial geniculate body (MGB), inferior colliculus (IC), superior olivary complex (SOC), cochlear nuclei (CN), and medial nuclei of the trapezoid body (MNTB). We sought to assess whether and when amyloid was present in these regions in 5xFAD mice and APP/PS1 mice.

**Figure 5:**
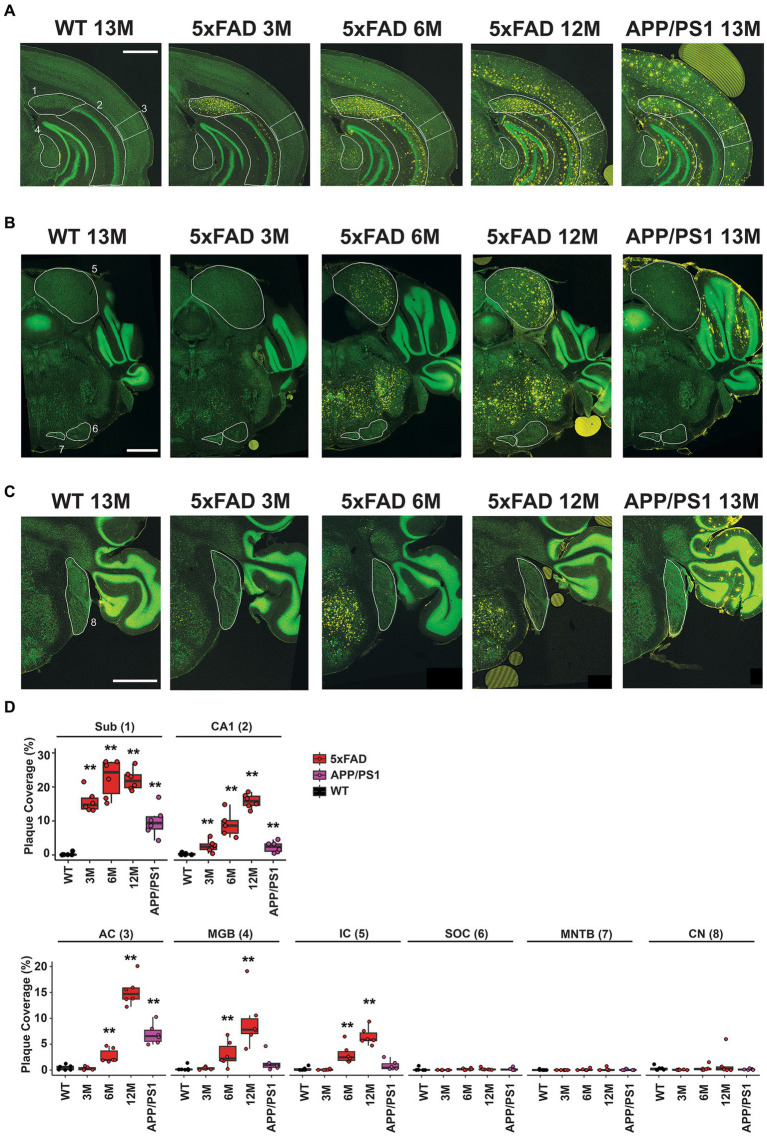
Differential amyloid plaque accumulation in the central auditory pathway of 5xFAD mice compared to APP/PS1 mice correlates temporally with the onset of central gain. Coronal sections from 13 M wild-type, 3 M 5xFAD, 6 M 5xFAD, 12 M 5xFAD, and 13 M APP/PS1 mice harboring important auditory nuclei were stained with anti-amyloid antibody (**A–C**, 5× images, yellow) and counterstained with Neurotrace to reveal nuclei (**A–C**, green). **(A)** Sections containing hippocampal subiculum (1, Sub), CA1 region (2), auditory cortex (3, AC) and the medial geniculate body (4, MGB) are compared. Regions are indicated with white outlines. **(B)** Sections containing the inferior colliculus (5, IC) the superior olivary complex (6, SOC) and the medial nucleus of the trapezoid body (7, MNTB) are compared. **(C)** Sections containing the cochlear nucleus (8, CN) are compared. All scale bars are 1,000 microns. **(D)** The percent area for each region that is covered with plaque is displayed (*n* = 6). Data are presented by modified box plots with jitter points represent individual animals. Asterisks denote the significant differences between genotypes: no significance (n.s.). *p* ≥ 0.05; *p < 0.05; **p < 0.01; and ***p < 0.001.

Coronal sections approximately −3.27 mm from Bregma reveal the subiculum and CA1 regions of the hippocampus, the AC, and the MGB for 13 M wild-type mice, 5xFAD mice at 3 M, 6 M, and 12 M, and APP/PS1 mice at 13 M (Figure 5A). Regions of interest are indicated in white and labeled 1–4, respectively, on the first panel. Plaque deposition, revealed by an anti-amyloid antibody, is observed for both the subiculum (Figure 5A, yellow, 1) and the CA1 region (Figure 5A, yellow, 2) of the hippocampus in both transgenic strains at all ages, but not in wild-type mice. Plaque deposition in the AC (Figure 5A, yellow, 3) is evident for 6 M 5xFAD, 12 M 5xFAD, and 13 M APP/PS1. However, the MGB (Figure 5A, yellow, 4) has a different distribution in plaque accumulation in the 5xFAD beginning at 6 M, but is not apparent in 13 M APP/PS1 mice.

Coronal sections approximately −5.19 mm from Bregma reveal the IC, the SOC, and the MNTB. Similar to the MGB, a different plaque distribution is evident in the IC, which is part of the auditory thalamus (Figure 5B, yellow, 5). Plaque is evident at 6 M and 12 M for the 5xFAD, but not in 13 M APP/PS1 mice. In contrast, the SOC and MNTB do not accumulate plaque in either mouse strain (Figure 5B, yellow, 6 and 7). The dorsal and anterior cochlear nucleus, found approximately −6.11 mm from Bregma, similarly lacks plaque accumulation for either strain (Figure 5C, yellow, 8).

Plaque density in these regions of interest was quantified from 10x images using a custom CellProfiler pipeline (see Methods) and expressed as percent of area coverage (Figure 5D, *n* = 6 for each location, age, and genotype). Coverage for transgenic mice was compared to that of wild-type controls for each region, and significant differences were assessed with Wilcoxon tests (Supplementary Table S1). These calculations confirm that significant, differential levels of plaque deposition occur in the MGB and IC at the same time that increased central gain first manifests in 5xFAD mice. Significant levels of plaque are not present in these regions in 13 M APP/PS1 mice.

We replicated this study of plaque deposition by further analyzing the plaque density in the auditory pathway of 5xFAD male mice at 2, 3, 4, and 6 M using a publicly available dataset (Oblak et al., 2021). Here we also analyzed the anterior cingulate cortex (ACC). The anterior cingulate cortex (ACC) is one of the earliest affected regions in AD (Jung et al., 2019). The AC actively regulates the activity of the auditory brainstem *via* corticofugal modulation (Terreros and Delano, 2015). Activation of the ACC can directly modulate the activity of the AC (Sun et al., 2022) and thalamus (Bubb et al., 2021). Lastly, the ACC modulates event-related potential responses in humans (Crottaz-Herbette and Menon, 2006). In the AC, 5xFAD mice showed significant accumulation of amyloid plaques starting at 3 M (Supplementary Figure S2A). A similar accumulation onset was found in the ACC (Supplementary Figure S2B).

Significant plaque accumulation began at 3 M in the MGB (Supplementary Figure S2C), and the IC (Supplementary Figure S2D). At 4 M, significant plaque accumulation was observed in the SOC (Supplementary Figure S2E). However, the plaque density in this region appeared to be much lower than in the IC and MGB (for 6 M 5xFAD mice, mean = 0.0059% in SOC, mean = 1.10% in IC and mean = 1.90% in MGB). Note that for the C57/CBA F1 mice generated for this study, mean plaque accumulation for the IC and MGB at 6 M ranged from 2–3% and the plaque level was below our detection threshold in the SOC. Plaque accumulation was insignificant in the nuclei of lateral lemniscus (NLL), CN, and NTB at these ages (Supplementary Figures S2F–H, *p*-values in Supplementary Table S2).

Overall, we conclude that significant plaque accumulation occurs in central auditory pathway of 5xFAD mice at early stages. The time course of plaque accumulation in the auditory pathway coincided with the ABR abnormality observed in our 5xFAD mice. The amyloid plaque also showed differential distribution in the auditory pathway: it accumulated at cortical levels and the upper auditory brainstem, but not lower levels of brainstem such as the CN. The plaque distribution appeared consistent with the change in the ABR waveform: wave IV has been proposed to originate from activities in the IC (Land et al., 2016; Möhrle et al., 2016). Whether the abnormal central activity arose from changes within the brainstem or the altered stimulation at cortical levels requires further investigation.

## 4. Discussion

In the present study, we evaluated central auditory activity in the transgenic AD mouse models 5xFAD and APP/PS1. ABR waveform analysis revealed increased central gain in 5xFAD mice, in contrast to reduced central gain in APP/PS1 mice. In addition, 5xFAD mice had more pronounced hearing loss than APP/PS1 mice. Analysis on the aging effect in 5xFAD mice showed that their central gain increase developed prior to other changes in hearing metrics, including wave I amplitude reduction, increased hearing threshold and the loss of hearing tone pips in noise. Pharmacologically facilitating acetylcholine transmission attenuated the increased central gain, indicating that this change may originate from a cholinergic deficit. This was also the first time that a deficit for hearing in noise, one of the core symptoms of CAPD/APD was observed in 5xFAD mice (Ouchi et al., 2015; Iliadou et al., 2017b). Finally, using a public database, we determined that plaque deposition significantly increases in auditory cortical regions, the auditory thalamus (MGB), and superior regions of the auditory brainstem prior to the central gain increase. These increases were not seen in APP/PS1 mice, where increased central gain was not observed. These experiments add to the growing body of literature consistent with the interpretation that plaque deposition in mouse models of amyloidosis correlate with central auditory processing disorder.

### 4.1. Potential mechanisms linking central auditory gain and AD pathology

In this study, we observed a central gain increase in 5xFAD mice (Figures 2, 3), which is commonly interpreted as a reflection of central hyperactivity. This enhanced neural activity can develop in multiple auditory structures, including the AC, IC, and CN (Salvi et al., 2000; Chambers et al., 2016; Schrode et al., 2018; Shaheen and Liberman, 2018; Parthasarathy et al., 2019; Resnik and Polley, 2021). This phenomenon has been attributed to decreased inhibition in those structures, with multiple inhibitory neurotransmitters involved, including GABA and acetylcholine (Caspary et al., 1995; Wang et al., 2002; Dong et al., 2010; Middleton et al., 2011; Balaram et al., 2019; Ma et al., 2020; Shao et al., 2021). In AD, inhibitory deficits have been identified as a consequence of AD symptoms in both clinical and animal model studies (Palop et al., 2007; Carvajal and Inestrosa, 2011; Verret et al., 2012; Hampel et al., 2018; Xu et al., 2020; Lauterborn et al., 2021). Specifically within the 5xFAD model, inhibition reduction is not uncommon (Flanigan et al., 2014; Yan et al., 2018). Taken together, we suggest that inhibitory signal disruption can be triggered by amyloid plaque deposition in the auditory pathway, leading to central hyperactivity and contributing to auditory processing disorder (APD). A recent review proposed inhibitory deficit as the link between central auditory processing disorder and AD (Pérez-González et al., 2022). Our study provides direct support for this hypothesis. Overall, inhibitory deficit is a possible mechanism by which increased central auditory gain was manifested in 5xFAD mice.

In patients, amyloid accumulation can be detected 10 years before the onset of clinical symptoms (Vermunt et al., 2019). Consistent with the reports regarding CAPDs in AD patients, AD-related pathological changes were identified in multiple sites of the central auditory system in AD patients (Baloyannis et al., 2009). Among those, the IC, MGB, and AC are sites where amyloid plaques and tau tangles were identified with the highest frequency (Ohm and Braak, 1989; Sinha et al., 1993). While all three of these sites accumulated plaque in the 5xFAD mouse model, we determined that only the AC accumulated significant levels of plaque in the APP/PS1 mouse, where central gain was not observed (Figure 5; Supplementary Table S1). Taken with studies on AD related inhibitory deficits, the central auditory hyperactivity in AD patients may be due to AD pathology in auditory pathway. Even though AD pathological changes were observed in both the cortices and brainstems of human AD patients, recent studies suggest that pathology in the brainstem might be a more robust predictor for AD dementia (Bidelman et al., 2017; Llano et al., 2021).

In addition to association with amyloid or tau pathology, studies in the auditory system suggest another possible link between AD pathological change and central hyperactivity: neuroinflammation. Recent studies demonstrated that neuroinflammation in the auditory system is associated with central hyperactivity and tinnitus (Shulman et al., 2021). Blocking neuroinflammation attenuated the excitation to inhibition imbalance induced by noise damage (Wang et al., 2019). Neuroinflammation is one of the primary pathological changes in AD (Leng and Edison, 2020), and may be present in the auditory system of AD patients, given that plaques and tangles were frequently identified in those regions (Ohm and Braak, 1989; Sinha et al., 1993). We speculate that neuroinflammation is an alternative origin for increased central gain in 5xFAD mice.

Central hyperactivity is frequently viewed as a consequence of reduced neuronal activity in the periphery (Parthasarathy et al., 2019). Here we showed that relative increases in auditory ERPs were present prior to wave I amplitude reduction and hearing loss in 5xFAD mice (Figure 2). This is in accordance with results by Iliadou et al. (2017a), who found that significantly reduced auditory temporal resolution associated with the transition between MCI and AD. Histological analysis showed that AD pathological changes were present in anatomical sites correlated in time with relative increases in auditory ERPs (Figure 5). Our data suggest that the enhanced central gain in 5xFAD mice is more likely a consequence of AD-related disruption in the central auditory system, instead of periphery degeneration.

### 4.2. Evidence for an inhibitory deficit as the link between auditory metric changes and AD

Acetylcholine is an inhibitory neurotransmitter that is widely distributed in the auditory pathway. We showed that relative increases in auditory ERPs in 5xFAD mice were attenuated by pharmacologically facilitating acetylcholine (Figure 4). This result suggests that an inhibitory deficit may have occurred in 5xFAD mice at 6 M. However, where and how that balance was disrupted requires further investigation.

In addition, we report a deficit for hearing tone pips in noise in 5xFAD mice (Figure 2). Hearing in noise deficits have been attributed to multiple auditory structures, including the AC (Resnik and Polley, 2021), IC (Wang et al., 2021), SOC (Lauer et al., 2022), as well as higher cortical areas (Tremblay et al., 2021). The SOC is one of the auditory nuclei that has been extensively studied regarding hearing in noise. The SOC modulates cochlear activity *via* efferent projections, mainly by releasing inhibitory neurotransmitters such as acetylcholine. A healthy auditory efferent system can readily suppress the effect of noise that is outside the frequency range of a signal (Lauer et al., 2022). Amyloid plaque accumulates in the AC, the MGB, and the IC in 5xFAD mice (Figure 5). It is possible that deficits for hearing tone pips in noise correlates with amyloid-related lesions in those regions. Alternatively, increased central gain may drive aberrant SOC activity, and eventually lead to hearing in noise deficits (Knudson et al., 2014; Dragicevic et al., 2015). Due to the administration of ketamine anesthetic, our hearing-in-noise test is independent of NMDA receptor function. Therefore, the deficit for hearing in noise in 5xFAD mice could be due to the cholinergic deficits. It remains unknown as to the relative contributions of the AD-related lesions in each of those regions to the deficit in 5xFAD mice.

### 4.3. ABR latency change in AD

Some studies have identified prolonged ABR latency in patients with AD or mild cognitive impairment (Harkins, 1981; Tachibana et al., 1996), while others did not find similar changes (Grimes et al., 1987; Kuskowski et al., 1991; Irimajiri et al., 2005). We showed that ABR peak latency shortened in the aged 5xFAD mice, when relative increases in auditory ERPs manifested (Figures 2, 3). This discovery is consistent with other findings regarding central hyperactivity in rodents (Möhrle et al., 2016; Qu et al., 2019). Even though the underlying mechanism still needs investigation, the reduced latency has been proposed as a direct consequence of neuronal gain and higher firing rates in neurons due to the decrease in inhibition in the auditory pathway (Möhrle et al., 2016).

### 4.4. Hearing loss in AD and the importance of these studies to patient care

The relationship between hearing loss and AD still remains unclear. Although a large population-based study in South Korea reported significant comorbidity between hearing loss and AD (Chang et al., 2020), studies in small cohorts have yielded mixed results: some studies report increased hearing thresholds in AD patients (Uhlmann et al., 1989; Strouse et al., 1995; Irimajiri et al., 2005; Gates et al., 2008), but others did not (Kurylo et al., 1993; Gates et al., 1995; Idrizbegovic et al., 2011). We showed that hearing loss severity varied between 5xFAD and APP/PS1 mice (Figure 3), which accumulate amyloid proteins at different rates (Lee and Han, 2013). Those results suggest that differential spatiotemporal amyloid accumulation can affect hearing loss progression in mice. Perhaps a similar differential in human disease could explain why some studies failed to identify significant hearing loss in AD and dementia patients.

Beyond abnormal hearing thresholds, we also observed a unique pattern of pathology progression: the ABR threshold increase was significant at lower frequencies before expanding to higher frequencies (Figure 2). This observation is similar to a previous study, which showed a greater mean difference at 8 and 16 kHz, than at 32 kHz between 5xFAD and WT mice (O’Leary et al., 2017). Given that the DPOAE thresholds remained normal when significant hearing loss manifested (Figure 2), the hearing loss in 5xFAD mice is likely due to auditory neuropathy, which is a common cause of presbycusis. However, in typical presbycusis, hearing loss is more prominent at high frequencies (Gates and Mills, 2005). Rather than having a synergistic effect with presbycusis, the pattern of hearing loss in 5xFAD mice suggests that amyloidosis contributes to hearing loss by a different underlying mechanism. We speculate that plaque deposition in the central auditory system plays a role in this hearing loss. It has been demonstrated in other animal models that lesions in the brainstem could cause hearing loss and accelerate cochlear aging (Liberman et al., 2014). A recent clinical study also showed that hearing loss and brainstem size are significantly correlated, but only in the context of AD (Llano et al., 2021). Others have similarly argued that AD-related damage to the central auditory cortices and their linked processing networks can drive central auditory processing disorders, especially by impacting temporal coordination (Johnson et al., 2021). Those studies suggest that auditory brainstem processing disorder can contribute to hearing loss in AD. However, the underlying mechanism of hearing loss in these transgenic AD mice needs further investigation.

In our studies, plaque deposition in the auditory midbrain, including the IC, correlated with a more rapid decline in hearing (Figures 3, 5), which in humans correlates with greater dementia progression (Thomson et al., 2017). We note that AD patients do accumulate plaques and tangles in the IC (Ohm and Braak, 1989; Sinha et al., 1993), and highlight here its importance for individuals with adult-onset hearing loss. The IC is important for lip-reading (Champoux et al., 2006), as it associates visual and auditory input in pre-conscious auditory processing (Gruters and Groh, 2012). We suggest that the existence of plaque in the auditory midbrain could precede a more rapid hearing loss and thus predict a more rapid decline. Further longitudinal studies are needed to determine if this correlation exists. If it does, such a biomarker would be important for patient care, provided that patients maintain hearing in adulthood. It would help patients to know when they would likely need a hearing aid. Moreover, caretakers who know that patients have difficulties with lip reading can take steps, like communicating medical results in quiet environments, to assist their patients in understanding speech. It could also prompt patients to arrange for greater levels of assistance or acquire a legal guardian. Lastly, changes in ABR metrics over time may be useful to stratify patient populations for later studies testing new treatments.

## 5. Limitations and caveats

One caveat of the present study is that our results are limited to male mice. In preliminary experiments (data not shown), female 5xFAD mice had different characteristics from their wild-type littermates from the earliest time points tested, leading to the interpretation that their hearing developed abnormally. Clinical studies show that more females than males have AD: almost two-thirds of Americans with AD are female (Alzheimer’s Association, 2021). Therefore, future investigations for female-specific changes are of important clinical value.

## Data availability statement

The raw data supporting the conclusions of this article will be made available by the authors, without undue reservation.

## Ethics statement

The animal study was reviewed and approved by University of Rochester’s Committee on Animal Resources University of Rochester Medical Center.

## Author contributions

DN: study conceptualization and design, methodology development, acquisition, analysis and interpretation of data, statistical analysis, first draft, review, and revision of the manuscript. JZ, HB, and DP-P: experimentation and editing. PN: experimentation, analysis, and editing. AK: technical, material support, and conceptualization. PW: study conceptualization, design, funding, material support, analysis, and revision of the manuscript. All authors read and approved the final manuscript.

## Funding

This work was funded by the National Institute of Health R01 DC014261-S1. The results of Supplementary Figure S2 are based on data obtained from the AD Knowledge Portal (https://adknowledgeportal.synapse.org/): The IU/JAX/UCI MODEL-AD Center was established with funding from The National Institute on Aging (U54 AG054345-01 and AG054349); Aging studies are also supported by the Nathan Shock Center of Excellence in the Basic Biology of Aging (NIH P30 AG0380770).

## Conflict of interest

The authors declare that the research was conducted in the absence of any commercial or financial relationships that could be construed as a potential conflict of interest.

## Publisher’s note

All claims expressed in this article are solely those of the authors and do not necessarily represent those of their affiliated organizations, or those of the publisher, the editors and the reviewers. Any product that may be evaluated in this article, or claim that may be made by its manufacturer, is not guaranteed or endorsed by the publisher.

## Footnotes

^1^https://github.com/PWhiteLab/FindPeaks
