# Corrigendum: Increased central auditory gain in 5xFAD Alzheimer's disease mice as an early biomarker candidate for Alzheimer's disease diagnosis

**DOI:** 10.3389/fnins.2023.1250244

**Published:** 2023-07-18

**Authors:** Daxiang Na, Jingyuan Zhang, Holly J. Beaulac, Dorota Piekna-Przybylska, Paige R. Nicklas, Amy E. Kiernan, Patricia M. White

**Affiliations:** ^1^Department of Biomedical Genetics, University of Rochester School of Medicine and Dentistry, Rochester, NY, United States; ^2^Department of Neuroscience, Ernest J. Del Monte Institute for Neuroscience, University of Rochester School of Medicine and Dentistry, Rochester, NY, United States; ^3^Department of Ophthalmology, University of Rochester, Rochester, NY, United States

**Keywords:** Alzheimer's disease, central auditory gain, hearing loss, auditory brainstem response, central auditory processing disorder, hearing in noise, inhibitory deficit

In the published article online, there was an error in **Figure 1** and **Figure 2** as published. The panels originally published as **Figure 1** are the data for **Figure 2**, and the panels originally published as **Figure 2** are the data for **Figure 1**. The corrected Figure 1 and its caption as well as the corrected Figure 2 and its caption appear below.

**Figure 1:**
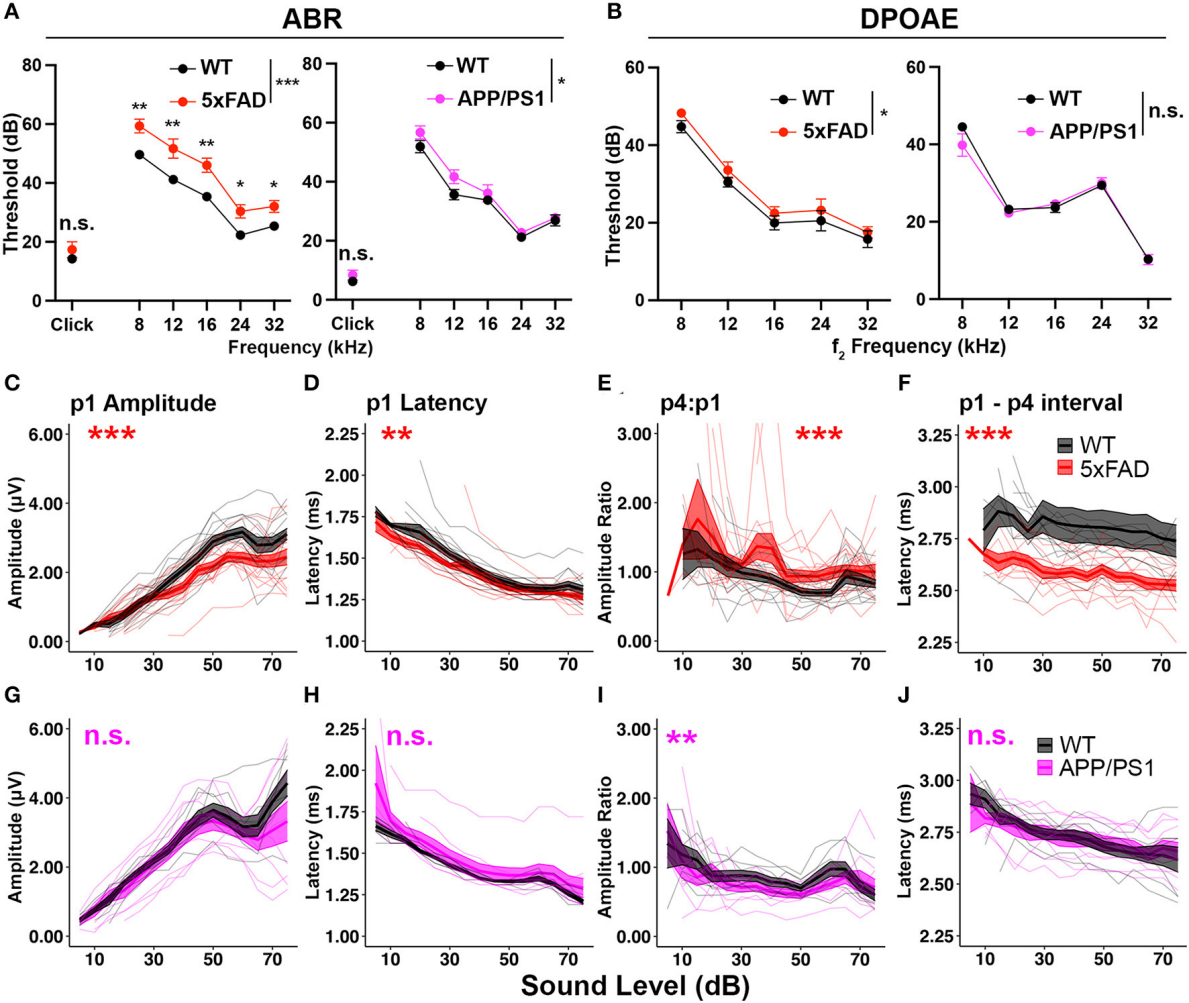
5xFAD transgenic mice have increased central gain and hearing loss severity. Auditory test results of **(A–F)** 5xFAD (red) at 12 months of age (12 M) (WT *n* = 13, 5xFAD *n* = 15), and **(G–J)** for APP/PS1 (magenta) at 13 M (WT *n* = 8, APP/PS1 *n* = 9). **(A–J)** Corresponding wild-type (WT) littermate data (black). **(A)** ABR and **(B)** DPOAE thresholds are expressed as the mean ± SEM. **(C–J)** Wave I (p1) amplitude, latency, wave IV to I amplitude ratio (p4:p1) and wave I to wave IV interpeak latency of click-evoked ABRs. Asterisks denote significant differences between genotypes: no significance (n.s.), *p* ≥ 0.05; *p < 0.05; **p < 0.01; and ***p < 0.001.

**Figure 2:**
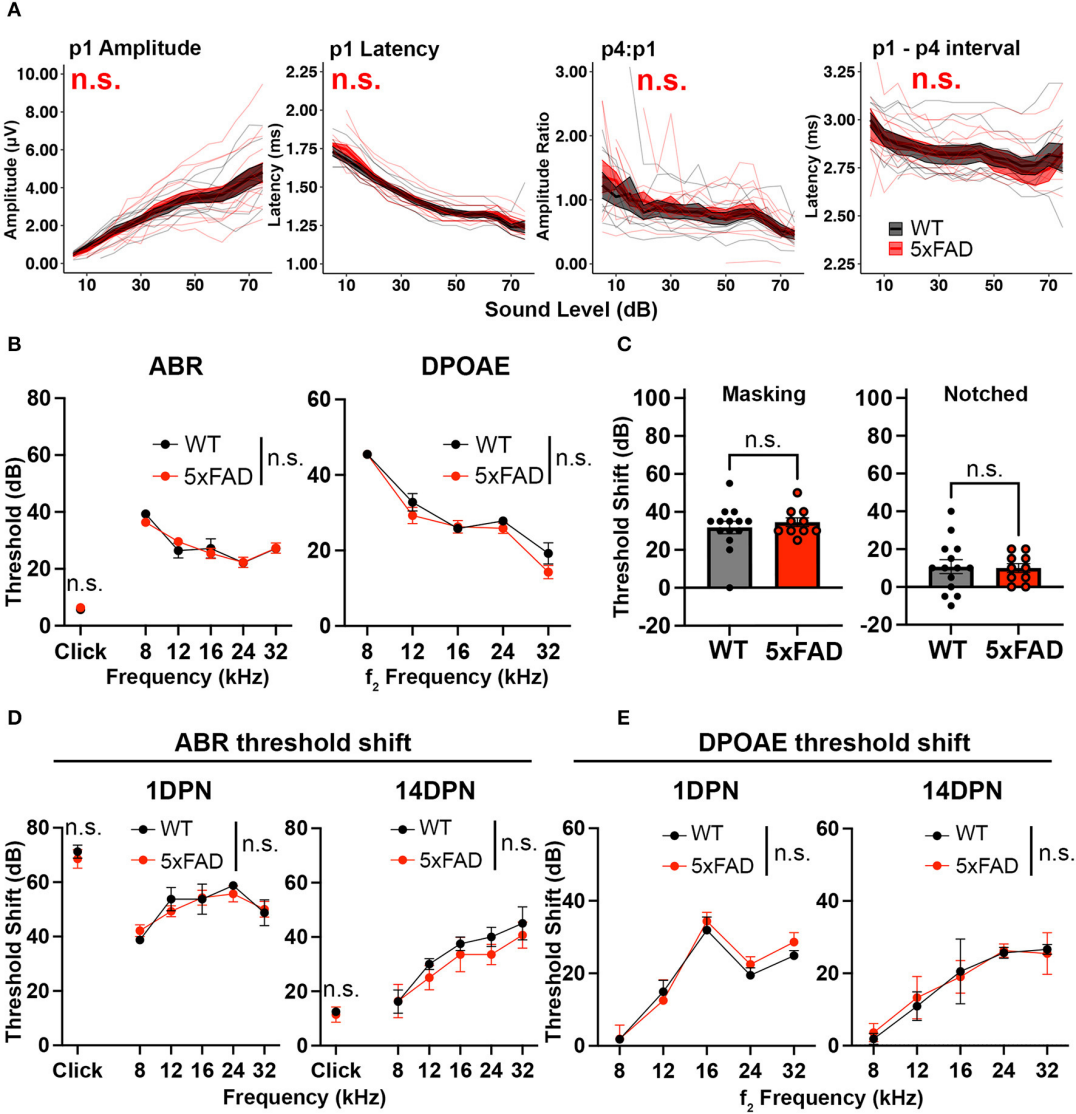
Auditory functions are normal in 5xFAD mice at an early stage of amyloid deposition. 5xFAD mice (red) and their WT littermates (black). **(A)** Wave I amplitude, latency, wave IV to I amplitude ratio, wave I to IV interpeak latency (from left to right) of click-evoked ABRs from 5xFAD mice (*n* = 14) and their WT littermates (*n* = 9) at 3 M. **(B–E)** Data are expressed as the mean ± SEM. **(B)** ABR (left) and DPOAE (right) thresholds from 5xFAD mice (*n* = 11) and their WT littermates (*n* = 7) at 3 M. **(C)** ABR threshold shift with masking and notched noise for 5xFAD mice (*n* = 10) and their WT littermates (*n* = 14) at 3 M. **(D)** ABR and **(E)** DPOAE threshold shifts at 1 day (1 DPN) and 14 days (14 DPN) post noise exposure for 5xFAD mice (*n* = 7) and their WT littermates (*n* = 4) at 3.5 M. No significance (n.s.).

In the published article, there was an error in the Funding statement. The authors incorrectly attributed funding from NIDCD R01 DC018660. The correct Funding statement appears below.

**FUNDING**

This work was funded by the National Institute of Health R01 DC014261-S1. The results of **Supplementary Figure S2** are based on data obtained from the AD Knowledge Portal (https://adknowledgeportal.synapse.org/): The IU/JAX/UCI MODEL-AD Center was established with funding from The National Institute on Aging (U54 AG054345-01 and AG054349); Aging studies are also supported by the Nathan Shock Center of Excellence in the Basic Biology of Aging (NIH P30 AG0380770).

The authors apologize for this error and state that this does not change the scientific conclusions of the article in any way. The original article has been updated.

