# Supplementary material for: Increased central auditory gain in 5xFAD Alzheimer’s disease mice as an early biomarker candidate for Alzheimer’s disease diagnosis
Source: Front Neurosci. 2023 May 26;17:1106570. doi: 10.3389/fnins.2023.1106570 (PMC10250613; doi:10.3389/fnins.2023.1106570)
Supplement: Supplementary file 1 [file Data_Sheet_1.PDF]

Supplementary table 1. P-values for plaque coverage for selected cortical regions in 5xFAD and APP/PS1 mice on C57/CBA hybrid background.

Regions	5xFAD_3M	5xFAD_6M	5xFAD_12M	APP/PS1_13M
**Subiculum**	0.002	0.002	0.002	0.002
**CA1**	0.004	0.004	0.002	0.004
**AC**	0.818	0.002	0.002	0.002
**MGB**	0.394	0.004	0.002	0.065
**IC**	0.937	0.002	0.002	0.093
**SOC**	0.2	0.226	0.092	0.375
**NTB**	0.405	0.599	0.599	0.599
**CN**	0.229	0.937	0.936	0.699

Plaque coverage values are displayed in Fig. 5 for 5xFAD sections at 3M, 6M, and 12M, as well as for 13M wild-type and 13M APP/PS1 (all n=6). Here the values for four transgenic samples are compared to WT with Wilcoxon to assess significance. Significant p-values ($p < 0.05$) are highlighted in yellow. Subiculum and CA1 are hippocampal regions. AC: auditory cortex; MGB: medial geniculate body; IC: inferior colliculus; SOC: superior olivary complex; NTB: nucleus of the trapezoid body; CN: cochlear nucleus.
