# Supplementary material for: Increased central auditory gain in 5xFAD Alzheimer’s disease mice as an early biomarker candidate for Alzheimer’s disease diagnosis
Source: Front Neurosci. 2023 May 26;17:1106570. doi: 10.3389/fnins.2023.1106570 (PMC10250613; doi:10.3389/fnins.2023.1106570)
Supplement: Supplementary file 2 [file Data_Sheet_2.PDF]

Supplementary Table 2. P-values for plaque coverage of selected cortical regions in 5xFAD mice on C57BL/6J background from the public database (see main text for details).

**Regions**	**3M vs 2M**	**4M vs 2M**	**6M vs 2M**
ACC	0.0006	0.0021	0.0048
AC	0.0018	0.0021	0.0024
MGB	0.0009	0.0021	0.0048
IC	0.0057	0.0021	0.0048
CN	>0.9999	>0.9999	>0.9999
SOC	0.6	0.0009	0.0024
NTB	>0.9999	>0.9999	0.105
NLL	>0.9999	>0.9999	0.0747

Plaque coverage areas shown in Supplementary Figure 2 were assessed for significance with Wilcoxon. As no wild-type data was available, percent coverage at 3M, 4M, and 6M were compared to 2M to assess accumulation. Highly significant values are highlighted in yellow. These results are similar to values obtained from C57/CBA mice with the exception of the SOC. See text for discussion. ACC: anterior cingulate cortex; AC: auditory cortex; MGB: medial geniculate body; IC: inferior colliculus; CN: cochlear nucleus; SOC: superior olivary complex; MNTB: nucleus of the trapezoid body; NLL: nuclei of lateral lemniscus.
