# Supplementary figures and images for: Increased central auditory gain in 5xFAD Alzheimer’s disease mice as an early biomarker candidate for Alzheimer’s disease diagnosis
Source: Front Neurosci. 2023 May 26;17:1106570. doi: 10.3389/fnins.2023.1106570 (PMC10250613; doi:10.3389/fnins.2023.1106570)

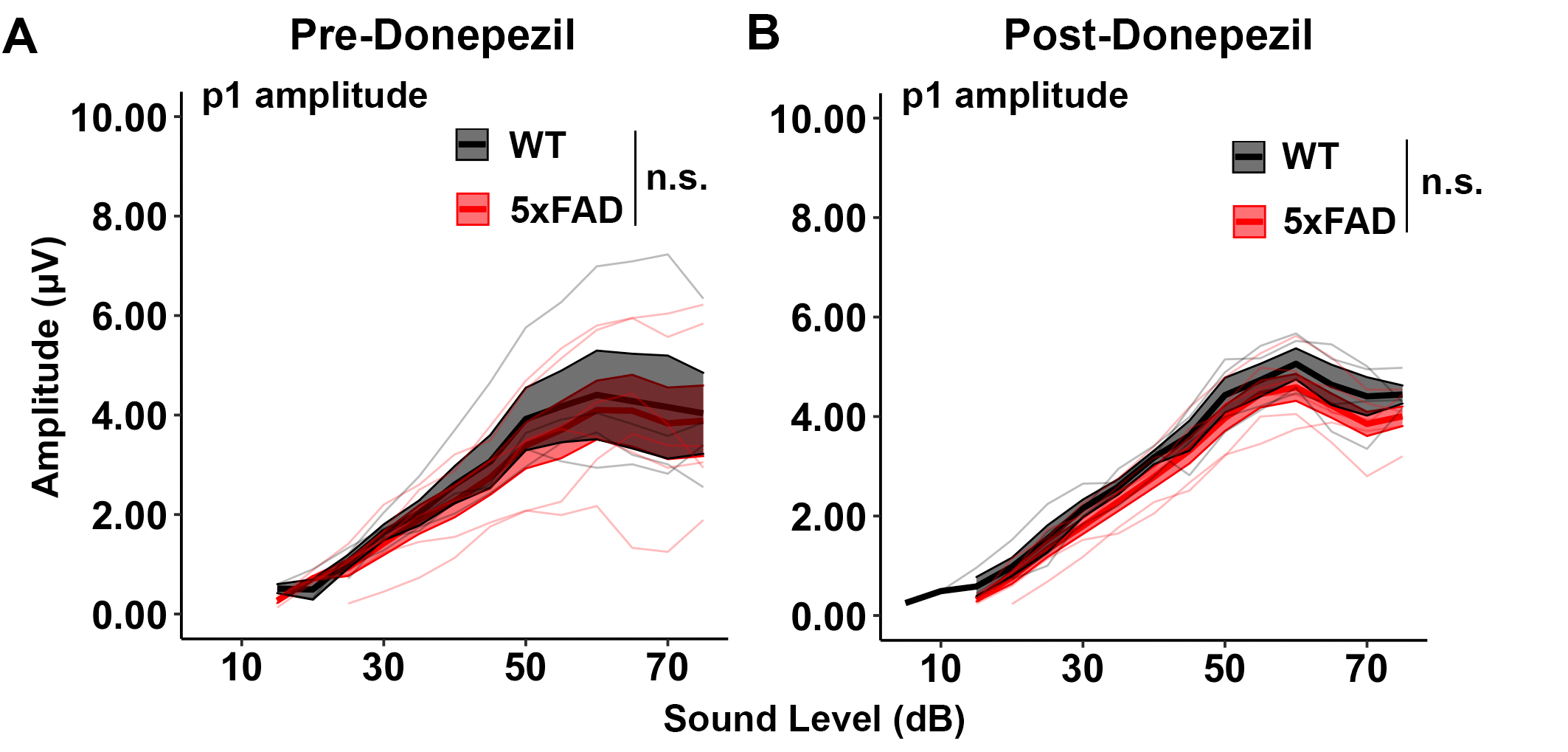

Supplement: Supplementary file 3 [file Image_1.TIF]

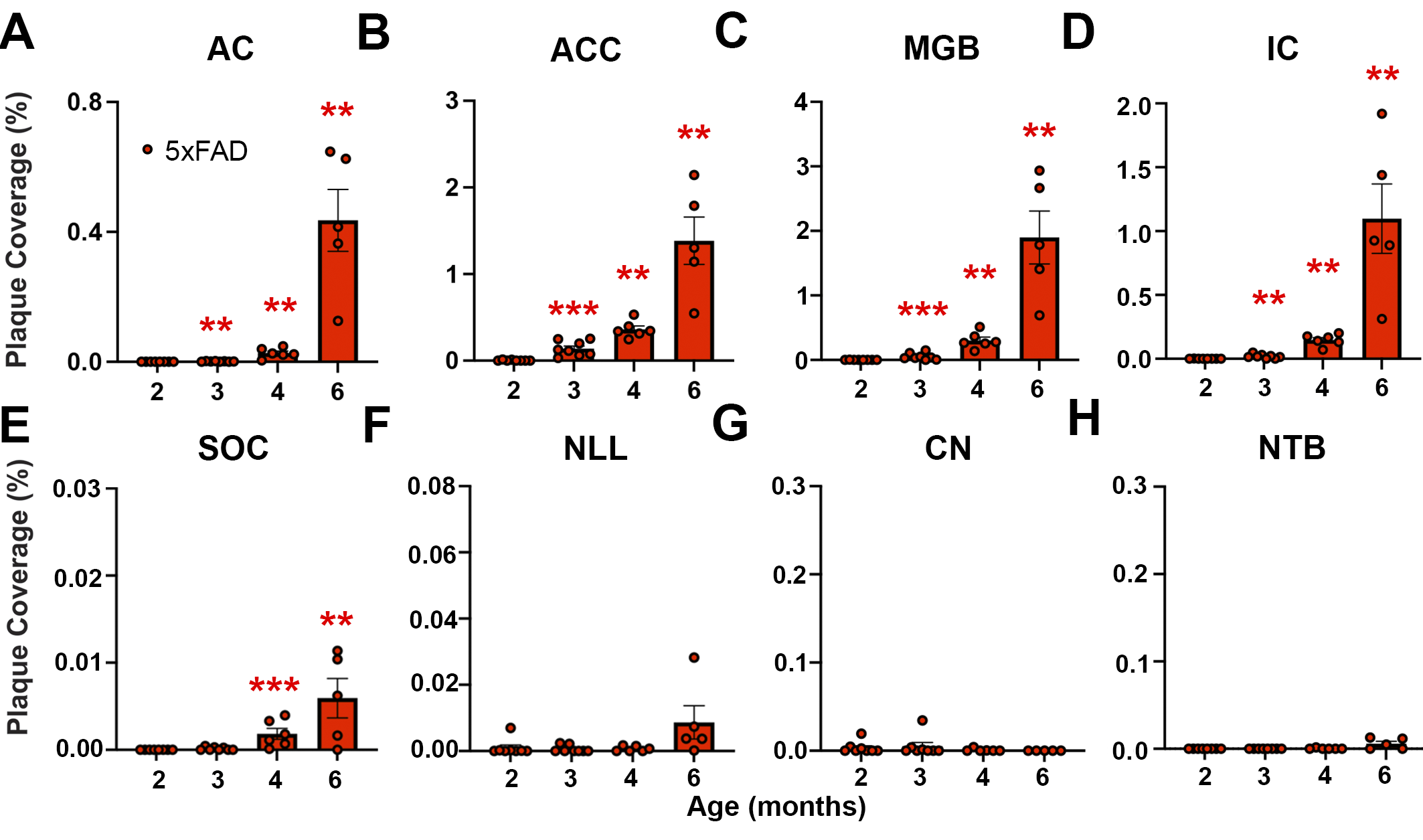

Supplement: Supplementary file 4 [file Image_2.TIF]
